# A Strategy to Prepare Primary Care Clinicians for Discussing Stopping Cancer Screening With Adults Older Than 75 Years

**DOI:** 10.1093/geroni/igaa027

**Published:** 2020-07-07

**Authors:** Mara A Schonberg, Maria Karamourtopoulos, Alicia R Jacobson, Gianna M Aliberti, Adlin Pinheiro, Alexander K Smith, Roger B Davis, Linnaea C Schuttner, Mary Beth Hamel

**Affiliations:** 1 Beth Israel Deaconess Medical Center, Harvard Medical School, Boston, Massachusetts; 2 Division of Geriatrics, Department of Medicine, University of California San Francisco; 3 Health Services Research & Development, VA Puget Sound Health Care System, Seattle, Washington; 4 Department of Medicine, University of Washington, Seattle

**Keywords:** Cancer prevention, Deimplementation, Overscreening

## Abstract

**Background and Objectives:**

Adults older than 75 years are overscreened for cancer, especially those with less than 10-year life expectancy. This study aimed to learn the effects of providing primary care providers (PCPs) with scripts for discussing stopping mammography and colorectal cancer (CRC) screening and with information on patient’s 10-year life expectancy on their patients’ intentions to be screened for these cancers.

**Research Design and Methods:**

Patient participants, identified via PCP appointment logs, completed a questionnaire pre- and postvisit. Primary care providers were given scripts for discussing stopping screening and information on patient’s 10-year life expectancy before these visits. Primary care providers completed a questionnaire at the end of the study. Patients and PCPs were asked about discussing stopping cancer screening and patient life expectancy. Patient screening intentions (1–15 Likert scale; lower scores suggest lower intentions) were compared pre- and postvisit using the Wilcoxon signed-rank test.

**Results:**

Ninety patients older than 75 years (47% of eligible patients reached by phone) from 45 PCPs participated. Patient mean age was 80.0 years (*SD* = 2.9), 43 (48%) were female, and mean life expectancy was 9.7 years (*SD* = 2.4). Thirty-seven PCPs (12 community-based) completed a questionnaire. Primary care providers found the scripts helpful (32 [89%]) and thought they would use them frequently (29 [81%]). Primary care providers also found patient life expectancy information helpful (35 [97%]). However, only 8 PCPs (22%) reported feeling comfortable discussing patient life expectancy. Patients’ intentions to undergo CRC screening (9.0 [*SD* = 5.3] to 6.5 [*SD* = 6.0], *p* < .0001) and mammography screening (12.9 [*SD* = 3.0] to 11.7 [*SD* = 4.9], *p* = .08) decreased from pre- to postvisit (significantly for CRC). Sixty-three percent of patients (54/86) were interested in discussing life expectancy with their PCP previsit and 56% (47/84) postvisit.

**Discussion and Implications:**

PCPs found scripts for discussing stopping cancer screening and information on patient life expectancy helpful. Possibly, as a result, their patients older than 75 years had lower intentions of being screened for CRC.

**Clinical Trials Registration Number:**

NCT03480282

**Translational Significance**: Guidelines recommend not screening older adults with less than 10-year life expectancy for cancer because the risk of harm significantly outweighs the chance of benefit for these patients; however, few primary care providers (PCPs) discuss stopping cancer screening with older adults. This study found that PCPs find information on their older patients’ 10-year life expectancy and scripts for discussing stopping cancer screening helpful and use of this intervention may lead to fewer older adults with short life expectancy and little chance of benefit intending to be screened. In addition, the study found that 56% of older adults are interested in discussing their 10-year life expectancy with their PCP; however, few PCPs reported feeling comfortable discussing 10-year life expectancy with older adults.

## Background and Objectives

The population of adults older than 75 years is rising and breast cancer and colorectal cancer (CRC) incidence increase with age (1). However, few randomized trials of mammography or CRC screening included older adults and the benefits of screening older adults are uncertain. A meta-analysis of the randomized trials of mammography screening and of fecal occult blood testing for CRC screening performed in the United States, Europe, and Canada found that it takes on average 10 years before 1 in 1000 adults may avoid dying from breast cancer (women only) or CRC as a result of screening (2). Due to this lag time to benefit and because there are harms to being screened, guidelines recommend discontinuing mammography and CRC screening for adults older than 75 years, especially those with less than 10-year life expectancy (3–8). Harms of cancer screening include anxiety, false-positive tests, complications from work-up of cancer, and overdiagnosis (detection of nonlethal tumors) leading to overtreatment (9). Despite these harms, approximately 30%–50% of U.S. adults 75 years or older undergo mammography (women only) and/or CRC screening, including many with less than 10-year life expectancy (10–13). The reasons for so many adults older than 75 years with little chance of benefit being screened include habit, lack of knowledge of the harms, concerns about cancer, and cultural norms that promote screening (14–16). Primary care providers (PCPs) recognize the need to talk to older adults about stopping cancer screening but find these conversations uncomfortable and feel ill-prepared (14,15).

Behavioral theory posits that having example scripts to inform challenging conversations increases clinicians’ capability to have these conversations (17). To help PCPs discuss stopping CRC and/or mammography screening with older adults, scripts were recently developed as a guide for PCPs to use when having these conversations (18). Three example scripts (available in the referenced article) were developed for discussing stopping breast cancer and CRC screening depending on the clinical scenario. The first script for each cancer is short and intended for PCPs to use when PCPs feel comfortable simply recommending a patient stop cancer screening due to their poor health. The second script for each cancer is slightly longer and suggests that PCPs inform adults older than 75 years that guidelines recommend that they discuss whether or not to continue having cancer screening tests with older adults. This script also provides a brief rationale as to why an older adult may want to stop cancer screening. The third script for each cancer gives example language for PCPs to use to discuss each of the benefits and harms of cancer screening tests to engage older adults in shared decision making. These scripts were developed with PCPs and older adults using qualitative methods in a research setting; therefore, it is not known whether PCPs seeing patients in practice would find them useful. The current study aimed to examine the effects of providing PCPs with these scripts before a routine visit with an adult aged 76–89 years. Primary care providers were also given information about their patients’ 10-year prognosis and life expectancy using validated measures (19–22). Investigators hypothesized that PCPs would find having the scripts and patient prognostic information helpful. As a result, PCPs would talk to their older patients about stopping cancer screening and fewer of their patients older than 75 years with little chance of benefit would intend to be screened for breast cancer (women only) or CRC.

## Research Design and Methods

### Design and Setting

From May 2018 to July 2019, the study investigated the effects of providing PCPs with scripts on discussing stopping mammography and CRC screening and with information on their patients’ 10-year prognosis and life expectancy on older adults’ cancer screening intentions. The study aimed to recruit 90 patients aged 76–89 years from 45 PCPs practicing at seven different Boston-area primary care practices including four community primary care practices, one community health center, one academic geriatrics practice, and one academic internal medicine practice that are all affiliated with one large academic medical center in Boston. These practices were selected due to their diversity and because they all use the same electronic medical records (EMRs). The medical director at each practice approved recruitment from their practice; no practice approached refused to participate. Beth Israel Deaconess Medical Center’s Institutional Review Board approved this study prior to data collection (BIDMC 2016P000244).

### Study Sample

A research assistant (RA) identified potentially eligible patients for this study by reviewing PCP appointment logs. Patients aged 76–89 years were eligible if they were English-speaking, not in hospice, had the capacity to participate, were screened with colonoscopy within 10 years or had a screening mammogram within 3 years (women only), and were scheduled to see their PCP in 3–12 weeks. Patients with a history of breast cancer and/or CRC or those whose last colonoscopy showed adenomas and women whose last mammogram was abnormal were excluded. Patients with a history of dementia (determined by review of medical record problem lists and then by communication with PCPs, or by a score of ≥10 [indicative of dementia] on the Short-Blessed Test administered via an eligibility questionnaire) (23) were also excluded. Patients whose medical records documented that they had stopped cancer screening and/or were intending to stop screening (as indicated by scores of 1–5 on a 15-point validated screening intentions scale assessed on an eligibility screen) were further excluded (24). Because the study aimed to include patients with approximately 10-year life expectancy, adults 76–79 years without a condition included in the Charlson comorbidity index (e.g., diabetes and heart failure) were excluded, because their estimated life expectancy is 15 years (25). The average life expectancy of adults older than 80 years is approximately 10 years (26). International Classification of Diseases-10 (ICD-10) codes and patient medical record problem lists were reviewed to identify patients aged 76–79 years with a Charlson comorbid condition.

### PCP Recruitment

Once the RA identified a potentially eligible patient via their medical records, she emailed his/her PCP to ask if the PCP was willing to receive example scripts for discussing stopping breast cancer and/or CRC and with life expectancy information for one to five of their patients older than 75 years. If so, the RA asked the PCP permission to contact their patients about the study. The RA also asked PCPs to exclude their patients with dementia or who were non-English-speaking. At the end of the study, from January to August 2019, investigators emailed PCPs whose patients participated to ask them to complete a study questionnaire. Primary care providers received a $50 incentive for completing a study questionnaire.

### Patient Recruitment

After obtaining PCP approval, the RA mailed patients a study informational letter with a number to call to opt out (the Institutional Review Board required that patients be offered an opportunity to opt out of being called by the RA). The RA called patients who did not opt out of initial telephone contact to assess their willingness to participate. After confirming eligibility via an eligibility questionnaire and obtaining verbal consent, the RA administered the previsit questionnaire. Patient participants received a $25 incentive.

### Data Collection

All study questionnaires are given in Supplementary Materials. The patients’ previsit questionnaire, completed a median of 18 days before a visit, assessed patients’ risk factors for 10-year mortality (19–22), their interest in talking to their PCP about their life expectancy, and their sociodemographics. The patients’ postvisit questionnaire, completed a median of 6 days after the visit, reassessed patients’ screening intentions (screening intentions were initially assessed on the patient eligibility questionnaire) (24) and their interest in talking to their PCP about their life expectancy. It also asked patients whether they discussed stopping screening with their PCP, what their PCP recommended, whether they discussed their life expectancy with their PCP, and included open-ended questions on their thoughts on these discussions. Primary care providers were asked whether they found the example scripts and/or the information on patient prognosis/life expectancy helpful and whether they used the information. Primary care providers were also asked to comment on their thoughts on discussing stopping cancer screening and patient life expectancy in open-ended questions. The RA reviewed notes from the study visit and excerpted deidentified text describing discussions of cancer screening or patient life expectancy.

### The Intervention

Within 3 days before a visit with a participating patient, the RA emailed PCPs a copy of the scripts for discussing stopping mammography and CRC screening. She also emailed PCPs a two-page report on their patient’s 10-year prognosis and life expectancy. The first page of the report presented patients’ 10-year prognosis based on the Lee–Schonberg index. This risk calculator, available on ePrognosis’s website, comprises two validated mortality indices (Lee/Schonberg) (19–22). The report presented the worse 10-year prognosis from the two indices and also noted whether the patient had less than or at least 10-year life expectancy based on the patient’s prognosis. As has been done previously, patients who had more than 50% 10-year mortality risk were considered to have less than 10-year life expectancy because life expectancy is the median survival of a population (19). The report also presented the list of risk factors included in these indices and noted which risk factors were considered in estimating the patient’s risk. The report’s second page included a copy of table 3 from the study of Cho et al. (22) which presents life expectancy for older adults based on U.S. life table data stratified by age (in 5-year age groups), sex, race (white/black/all), and comorbidity. Below Cho’s table, the report summarized patient life expectancy based on the table’s information and the patient’s characteristics. In the body of the email to PCPs, the RA included a two-line summary of their patient’s 10-year prognosis (based on Lee–Schonberg) and life expectancy (based on Cho) (19–22). There was 0.87 (95% CI 0.76–0.97) agreement (kappa statistic) in classifying patients as having less than 10-year life expectancy between the Lee and Schonberg indices and 0.66 (95% CI 0.54–0.78) agreement among the Lee, Schonberg, and Cho methods (Supplementary Figure S1 presents a Venn diagram of the agreement in 10-year life expectancy estimates using the Lee, Schonberg, and Cho methods within patients). Self-reported information from participants’ baseline questionnaires was used to estimate patient life expectancy; self-reported comorbidities were confirmed via patients’ medical records. There was only one case when a patient reported a medical problem (end-stage renal disease) that was not confirmed by the medical record and end-stage renal disease was not used to estimate patient life expectancy in that case.

Adults 76 years or older were included in this study even if their estimated life expectancy was at least 10 years because national guidelines recommend discussing stopping screening with these patients based on their age alone and all patients had close to 10-year life expectancy (3,4,7). Also, the third example script given to PCPs for each cancer provided language for PCPs to engage older adults in shared decision making about whether or not to continue screening. Guidelines, such as those from the American Cancer Society, that recommend screening older adults as long as their life expectancy is at least 10 years also encourage shared decision making around screening for adults older than 75 years (6,8). Therefore, it was appropriate to engage all patient participants in shared decision making about screening.

### Statistical Analysis

Our primary outcome of interest was the effect of the intervention on older adults’ intentions to be screened for CRC and a secondary outcome of interest was the effect of the intervention on older women’s intentions to be screened for breast cancer. To assess these outcomes, we compared patients’ pre- and postvisit intentions of obtaining screening tests. Because the observations were matched by the patient, we used the Wilcoxon signed-rank test, a nonparametric paired test. Because we anticipated a priori that at least 40 patient participants would be female, we estimated having 0.87 power to detect a 3.3 point mean difference in mammography screening intentions pre- and postvisit assuming a standard deviation of 5 and a within-pair correlation of 0.1. We estimated having greater power to show a decline in CRC screening intentions because both sexes may be screened for CRC. In secondary analyses, we examined whether the intervention had a differential effect on older adults’ screening intentions based on life expectancy (<10 vs ≥10 years from the Lee–Schonberg index). We also conducted thematic analyses to identify themes in participants’ open-ended comments and from text from visit notes (27). Two investigators reviewed all participants’ open-ended comments and the text from visit notes to identify themes. Discrepancies in themes identified by investigators were resolved by consensus. Statistical analyses were completed using SAS statistical software, version 9.4.

## Results

Supplementary Figure S2 demonstrates the PCP recruitment flow. Of 71 PCPs approached, 58 agreed to participate, 45 had at least one patient participate, and 37 completed the PCP questionnaire (Supplementary Table S1 presents PCP characteristics). Primary care provider participants were similar to nonparticipants (Supplementary Table S2). Supplementary Figure S3 demonstrates patient recruitment flow. Of 2,857 patient records reviewed, 2,263 were not eligible, 358 could not be reached, 35 wanted more time to consider whether or not to participate, 29 opted out of initial telephone contact, 71 declined participation, and 101 completed the previsit questionnaire. Of these 101 patients, 10 did not see their PCP during the study and one withdrew, leaving 90 patients. Patient mean age was 80 years (±3), 43 (48%) were female, 79 (88%) were non-Hispanic white, 37 (41%) were seen in community practices, and their mean life expectancy was 9.7 years (*SD* = 2.4) using Cho’s table 3; 52% had less than 10-year life expectancy using the Lee–Schonberg index. Patients who declined to participate were similar to participants based on age, race, sex, practice site, but had lower educational attainment (Supplementary Table S3). Table 1 presents patient characteristics.

**Table 1.** Patient Participant Characteristics

Patient Participants	*n* = 90
Age, mean (*SD*)	80.0 (2.9)
Recruitment site	
Boston academic, *n* (%)	53 (59)
Boston community, *n* (%)	37 (41)
Female gender	43 (48)
Non-Hispanic white, *n* (%)	79 (88)
Education	
High school or less, *n* (%)	15 (16)
Some college, *n* (%)	16 (18)
College degree or beyond, *n* (%)	59 (66)
Income*	
$35K or less, *n* (%)	12 (13)
>$35K to $65K, *n* (%)	11 (12)
>$65K or higher, *n* (%)	45 (50)
Declined to answer, *n* (%)	22 (24)
Currently married, *n* (%)	65 (72)
10-year life expectancy from Lee–Schonberg index^†^	
≥10-year life expectancy, *n* (%)	43 (48)
<10-year life expectancy, *n* (%)	47 (52)
10-year life expectancy from Cho method^‡^ and mean life expectancy (*SD*)	9.7 years (2.4)
≥ 10-year life expectancy, *n* (%)	49 (54)
< 10-year life expectancy, *n* (%)	41 (46)
Difficulty with understanding written medical information, *n* (%)^§^	15 (17)
≥1 First-degree family history of colorectal cancer	11 (12)
≥1 First-degree female history of breast cancer (*n* = 43 women only)	6 (14)
Last colorectal cancer screening*	
<5 years ago, *n* (%)	50 (56)
≥5 years but <10 years, *n* (%)	40 (44)
Number of colonoscopies from the medical records	
1, *n* (%)	9 (10)
2, *n* (%)	35 (39)
3 or more, *n* (%)	46 (51)
History of mammography use from the medical records	*n* = 43
Every year, *n* (%)	37 (86)
Every other year, *n* (%)	6 (14)
Years with PCP, mean (*SD*)	9.2 (6.8)
I have complete trust in my primary care doctor	89 (99)

*Note:* PCP = primary care provider.

*Proportions do not add to 100% due to rounding.

^†^We used the lower life expectancy from either the Lee or Schonberg mortality index. Schonberg index: Scores ranged from 3 to 23. Scores ≥10 are associated with a more than 50% chance of 10-year mortality. Thus, adults who score ≥10 are estimated to have less than 10-year life expectancy. Lee mortality index: Scores ranged from 4 to 12. Scores ≥8 are associated with a more than 50% chance of 10-year mortality. Thus, adults who score ≥8 are estimated to have less than 10-year life expectancy (19–21).

^‡^Cho et al. (22) estimated life expectancy using U.S. life table data stratified by sex, age (in 5-year age groups), race (white, black, all), and adjusting for comorbidity. Participant life expectancy ranged from 4.8 to 15.3 years using Cho et al.’s table 3.

^§^Health literacy was assessed by reporting difficulty with filling out medical forms by oneself, difficulty learning about one’s medical condition due to difficulty understanding written information or needing family/friend to help read hospital materials (28).

### Patient CRC Screening Intentions

Table 2 presents the intervention’s effects on patients’ cancer screening intentions and their perceptions of discussions around stopping cancer screening and/or prognosis. Sixty-three patients (71%, 63/89) reported discussing CRC screening with their PCP during the visit, of which 49% (31/63) specifically reported that their PCP discussed stopping CRC screening. Of the 63 patients who reported discussing CRC screening, 23 (37%) thought their PCP recommended stopping screening (57% [16/28] with <10-year life expectancy vs 20% [7/35] with ≥10-year life expectancy, *p* = .01), 28 (44%) thought their PCP recommended continuing screening, and 13 (21%) reported that their PCP said it was the patient’s decision. Nineteen (21%) patients reported that their PCP discussed CRC screening’s harms. Overall, mean CRC screening intentions declined (9.0 [*SD* = 5.3] to 6.5 [*SD* = 6.0], *p* < .0001), and there were no significant differences in decline in CRC screening intentions by sex (*p* = .60). Specifically, 39 patients (43%) reported lower CRC screening intentions postvisit (49% [22/45] of those with <10-year life expectancy vs 40% [17/43] of those with ≥10-year life expectancy, *p* = .60; Supplementary Table S4). Other patients (*n* = 39) did not change their screening intentions or had increased intentions (*n* = 10).

**Table 2.** Patient Perceptions of Discussions About Stopping Cancer Screening and/or Prognosis

Outcomes	Baseline, *n* = 90	Follow up, *n* = 90	*p* Value
*Colorectal cancer screening (CRC)*			
Talked to PCP about stopping CRC screening at study visit,* *n* (%)		31 (34)	
PCP talked about the downsides of colonoscopies/stool tests, *n* (%)		19 (21)	
Missing		1 (1)	
What did your PCP recommend?			
Continue having colonoscopies/stool tests		23 (26)	
Have one more colonoscopy/stool test then stop		5 (6)	
Stop having colonoscopies/stool tests		23 (26)	
Made no recommendation, said it was my choice		12 (13)	
We did not discuss colonoscopies or stool tests		26 (29)	
Missing		1 (1)	
Intentions to be screened for CRC,^†^ overall, mean (*SD*)	9.0 (5.3)	6.5 (6.0)	<.0001
Intentions moved toward CRC screening, *n* (%)		10 (11)	<.0001
Intentions stayed the same, *n* (%)		39 (43)	
Intentions moved away from CRC screening, *n* (%)		39 (43)	
Which screening test do you plan to have?			
Colonoscopy		57 (63)	
Other		3 (3)	
Do not plan to have CRC screening		29 (32)	
*Mammography screening (women only)*		*n* = 43, women only	
Talked to PCP about stopping mammography screening at study visit, *n* (%)		17 (40)	
PCP talked about downsides of mammography screening, *n* (%)		6 (14)	
Missing		1 (2)	
What did your PCP recommend?			
Continue having mammograms		11 (26)	
Have another mammogram then stop		3 (7)	
Stop having mammograms		6 (14)	
Made no recommendation, said it was my choice		12 (28)	
We did not discuss mammograms		11 (26)	
Intentions to be screened with mammography,^§^ overall, mean (*SD*)	12.9 (3.0)	11.7 (4.9)	.08
Intentions moved towards mammography, *n* (%)		2 (4)	.07
Intentions stayed the same, *n* (%)		30 (70)	
Intentions moved away from mammography, *n* (%)		9 (21)	
When do you plan to get your next mammogram?			
I do not plan on getting another mammogram, *n* (%)	0	6 (14)	<.0001
In the next year, *n* (%)	37 (86)	20 (46)	
>1 year from now but <2 years from now, *n* (%)	3 (7)	2 (5)	
>2 years from now, *n* (%)	1 (2)	0	
Plans to get another but not sure when, *n* (%)	1 (2)	14 (32)	
Missing, *n* (%)	1 (2)	1 (2)	
Intentions to be screened for CRC among women only,^†^ overall, mean (*SD*)	9.9 (5.0)	7.9 (5.9)	.005
*Life expectancy/prognosis*		*n* = 90	
Talked to PCP about how long I may have to live at study visit, *n* (%)		24 (27)	
My preference for prognostic/life expectancy information:	—		
As a range, for example, “5–10 years,” *n* (%)	—	22 (24)	
As a probability, i.e., “50/50 chance of living 10 years,” *n* (%)	—	21 (23)	
As a number, for example, “10 years,” *n* (%)	—	18 (20)	
No preference, *n* (%)		29 (32)	
Are you interested in talking to your doctor about how long you may have to live?			.14
Not at all, *n* (%)	32 (36)	37 (41)	
A little, *n* (%)	9 (10)	4 (4)	
Somewhat, *n* (%)	14 (16)	16 (18)	
A great deal, *n* (%)	31 (34)	27 (30)	
Missing, *n* (%)	4 (4)	6 (7)	
I would want information on how long I may have to live in deciding whether to get tested for cancer, *n* (%)		36 (40)	
Missing, *n* (%)		11 (12)	
I have thought about how long I may have to live, *n* (%)		69 (77)	
Missing, *n* (%)		1 (1)	
I have talked with my children/family about how long I may have to live, *n* (%)		47 (52)	
*From the medical records*		*n* = 90	
PCP talked about cancer screening, *n* (%)		52 (58)	
PCP talked about mammography (*n* = 43), *n* (%)		25 (58)	
PCP talked about prognosis/life expectancy, *n* (%)		5 (6)	

*Note:* PCP = primary care provider.

*Proportions do not add to 100% due to rounding.

^†^Intentions to be screened for CRC—1 (I will not have a colonoscopy/stool test in the next few years) to 8 (undecided) to 15 (I will have a colonoscopy/stool test in the next few years).

^§^Intentions to be screened for breast cancer with mammography—1 (I will not have a mammogram in the next year) to 8 (undecided) to 15 (I will have a mammogram in the next year).

### Patient Mammography Screening Intentions

The intervention was associated with similar but more muted effects on women’s (*n* = 43) mammography screening intentions (Table 2). Thirty-two women (74%) reported discussing mammography screening at the visit; of these, 53% (17) reported that their PCP specifically discussed stopping screening. Of the 32 who reported discussing mammography, six (19%) thought their PCP recommended stopping screening, three (9%) thought their PCP recommended having one more mammogram then stopping, 11 (34%) thought their PCP recommended continuing screening, and 12 (38%) reported that their PCP said it was the patient’s choice. Six (14%) women reported that their PCP discussed mammography’s harms. On average, mammography screening intentions tended to decline (12.9 [*SD* = 3.0] to 11.7 [*SD* = 4.9], *p* = .08). Nine women (22%) had lower mammography screening intentions postvisit (29% [4/14] with <10-year life expectancy vs 19% [5/27] with ≥10-year life expectancy, *p* = .51). Other patients did not change their screening intentions (*n* = 30) or their intentions increased (*n* = 2). While many women continued to intend to be screened, postvisit fewer intended to be screened in the next year (90% [37/41] vs 49% [20/41], *p* < .0001). Screening intentions were higher for breast cancer than CRC both pre- and postvisit.

Overall, 40 patients reported discussing stopping either CRC or breast cancer screening with their PCP (43% [17] with <10-year life expectancy). Of these, none described a negative experience. Many commented that they felt comfortable talking to their PCP about anything: “I have known him for many years so we can talk about anything.” Also, while these patients reported discussing stopping screening with their PCP many still intended to be screened. “I am entitled to have a colonoscopy or mammogram, no one says that I have to stop.” Others described moving to less aggressive screening as a result of the discussion, “We decided to skip this year and revisit at a later time.”

### Patient Perspectives on Discussing Life Expectancy When Discussing Screening With PCPs

Overall, 47 patients (56%) reported postvisit being at least a little interested in discussing life expectancy with their PCP, 36 (46% [36/79]) thought life expectancy information would be helpful when deciding on cancer screening, and 24 (27%) reported discussing their life expectancy with their PCP (including three who reported no interest in these discussions). Sixty-nine patients (78%) reported thinking about their life expectancy on their own and 47 (52%) reported having talked to the family about their life expectancy. Supplementary Table S5 summarizes the themes identified in patients’ open-ended comments on discussing stopping cancer screening and their life expectancy with their PCPs. While many patients were enthusiastic about cancer screening “I will do whatever I can to stop myself from getting cancer,” they also expressed wanting high-value care. “If she could show me that not having one was better than having one I would trust that.”

Patients had varying views on whether PCPs should discuss patient life expectancy with older adults, with some saying “I don’t care to discuss it at all” and others saying “it is a good idea.” Many felt it was impossible to predict someone’s life expectancy. Several patients noted that while they would not feel comfortable bringing up their life expectancy on their own they would appreciate their PCP bringing up this topic. “I never would have said anything but I had been wondering how things are going.”

Of the 24 patients who reported discussing their life expectancy with their PCP at the visit, none reported having a negative experience. However, patients were conflicted regarding the value of this information. Some found the information helpful: “I think talking about death is always helpful,” whereas others felt that it is impossible to really know when someone will die: “It’s an interesting conversation but it doesn’t tell me very much, it’s just a guess and there is no validity to it.”

### PCP Perspectives on Discussing Stopping Cancer Screening and Life Expectancy

Thirty-two (89%) PCPs found the scripts for discussing stopping screening helpful and 29 (81%) said they would use them frequently (Table 3). Primary care providers documented in patient medical records that their patients’ risk factors, age, health, preferences, and prior screening results affected their screening recommendations and patients’ decisions (Supplementary Table S6). Primary care providers also documented deferring these discussions if screening was up-to-date. Thirty-five PCPs (97%) reported that they found patient life expectancy information helpful; 29 (78%) reported using life expectancy information to talk to at least one patient about stopping screening and 17 (46%) reported using the information to talk to at least one patient about their life expectancy. While PCPs felt that life expectancy information would help their patients make more informed decisions (29/35, 83%) and to plan for their future (*n* = 33, 89%), only eight PCPs (22%) felt comfortable talking to patients about their life expectancy. Most PCPs (*n* = 32, 87%) reported that they would like patient prognostic/life expectancy information in EMRs.

**Table 3.** Primary Care Provider (PCP) Perspectives on the Scripts for Discussing Stopping Cancer Screening and Prognostic Information

PCP Perspectives	*n* = 37
I found the scripts for discussing stopping mammography/colonoscopy helpful*	
Agree, *n* (%)	32 (86)
Neutral/Disagree, *n* (%)	4 (10)
Missing, *n* (%)	1 (3)
I would use the scripts frequently*	
Agree, *n* (%)	29 (78)
Neutral/Disagree, *n* (%)	7 (19)
Missing, *n* (%)	1 (3)
I would recommend the scripts to my colleagues	
Agree, *n* (%)	28 (76)
Neutral, *n* (%)	7 (19)
Missing, *n* (%)	2 (5)
I found the information on my patient’s prognosis/life expectancy*	
Very helpful, *n* (%)	11 (30)
Somewhat helpful, *n* (%)	21 (57)
A little helpful, *n* (%)	3 (8)
Not helpful, *n* (%)	1 (3)
Missing, *n* (%)	1 (3)
Was the life expectancy information accurate from your perspective?	
Very accurate, *n* (%)	24 (65)
Somewhat accurate, *n* (%)	12 (32)
Missing, *n* (%)	1 (3)
I used the prognostic information to talk to patients about stopping cancer screening, *n* (%)	29 (78)
I used the information to talk to patients on how long they may have to live, *n* (%)	17 (46)
I am uncomfortable talking to my older patients about how long they may have to live, *n* (%)	82 (78)
I would like prognostic information in the electronic medical record,* *n* (%)	32 (87)
Providing my older patients with information about how long they may have to live would result in my patients making more informed decisions about their medical care*	
Agree, *n* (%)	29 (78)
Neutral, *n* (%)	6 (16)
Missing, *n* (%)	2 (5)
I prefer to talk to my older patients about how long they may have to live because it may help them plan for their future	
Agree, *n* (%)	33 (89)
Neutral, *n* (%)	4 (11)
I prefer to talk to my older patients about how long they may have to live because it may help them with medical decisions	
Agree, *n* (%)	27 (73)
Neutral, *n* (%)	9 (24)
Missing, *n* (%)	1 (3)
I prefer not to talk to my older patients about how long they may have to live because I do not want them to think I have given up on them	
Agree, *n* (%)	17 (46)
Neutral/Disagree, *n* (%)	20 (54)
I prefer not to talk to my older patients about how long they may have to live because it is impossible to know how long someone may live	
Agree, *n* (%)	12 (32)
Neutral/Disagree, *n* (%)	25 (68)

*Proportions do not add to 100% due to rounding.

Table 4 displays themes in PCPs’ open-ended comments. Primary care providers expressed that discussing stopping cancer screening with older adults is important, is easier with high-literacy patients, and with patients with whom they have a strong relationship. Primary care providers found the scripts on discussing stopping cancer screening helpful and did not recommend revisions. “They [the scripts] make it a lot easier to discuss a topic that doctors are usually hesitant to bring up.” PCPs also recommended the scripts be included in EMRs for easy access. “I wonder about a link in the EMR because otherwise it would be difficult to remember them.”

**Table 4.** Primary Care Provider (PCP) Themes on Discussing Stopping Screening and Prognosis With Adults Older Than 75 Years (*n* = 37)

PCP Themes	Example Quotes
*Discussing stopping cancer screening*	
Discussing stopping cancer screening is important	“I think it’s important. Most patients are ready to stop, in my experience.”
Easier with higher literacy patient	“Generally gone well for most patients, particularly those who are higher health literacy and can understand the risks/benefits better.”
Helpful to have a strong doctor–patient relationship	“Having a relationship with the patient first makes these conversations easier.”
The scripts were helpful	“This has been helpful and makes patients feel at ease with aging and what tests are needed or not needed.”
*Discussing life expectancy*	
Discussing life expectancy is important but uncomfortable	“Even though I feel uncomfortable talking about their life expectancy, I believe there are many benefits for doing so.” “I recognized the benefit but still feel the discomfort.’
Life expectancy easier to discuss in the context of a decision	“Easier to discuss in terms of a particular decision (screening, etc.) rather than giving an actuarial estimate outside of that type of decision.”
Depends on the patient	“It is very patient dependent—depends on personality and how significant their medical conditions are.” “This is hard but often patients are relieved to discuss it.”
Practice needed to discuss	“I think if I start doing it more routinely, then it will become easier.”
Need to normalize it	“Normalizing it—having a statement that we try to talk about all of our patients about this.”
Takes time	“It would be time-consuming to do this for all patients without more support or time”
Estimates are helpful	“The life expectancy/prognosis information should be given to all older patients yearly.” “The information is useful.” “It would be easier to make medical decisions.”
Views vary on whether life expectancy or prognosis more helpful	“I don’t like to tell patients you are expected to live for 5 more years, and find it easier to tell them the prognosis.” “I prefer to receive both types of estimates.” “Having both is helpful.”
Helpful to have life expectancy/prognosis in EMR with caveats	“It would be easier to make medical decisions.” “Would be additional useful information as long as there was a caveat about accuracy.” “I wouldn’t want a patient to see it if we never spoke about it.”

*Note:* EMR = electronic medical record.

In regard to discussing life expectancy with older adults, PCPs felt that having estimates of patient life expectancy was helpful for medical decision making and that it would be helpful to have these estimates in patients’ EMRs; however, some were concerned with patients seeing this information on their own. Primary care providers had varying views on whether older adults want to discuss their life expectancy with their PCP. For example, one PCP commented “I think the life expectancy information should be given to all patients yearly,” whereas another felt that “patients do not want to be told how long their life expectancy is.” PCPs further noted that discussing life expectancy could be time-consuming but felt it may become easier with practice, “if I start doing it more routinely, it will become easier.” Several PCPs commented that even though they felt discussing life expectancy was uncomfortable they felt it was crucial. “Even though I feel uncomfortable talking about their life expectancy, I believe there are many benefits for doing so.” Some also recognized that some patients may be more comfortable with this topic than PCPs: “I imagine that patients are probably more comfortable talking about it than some providers.”

## Discussion and Implications

Providing PCPs with their patients’ estimated 10-year prognosis/life expectancy and scripts for discussing stopping cancer screening before a visit was associated with fewer adults older than 75 years intending to undergo CRC screening and to fewer women older than 75 years intending to be screened with mammography in the next year. Primary care providers found the scripts and patient prognostic information helpful and many reported using the information to discuss stopping cancer screening. Patients with less than 10-year life expectancy were more likely to report that their PCP recommended stopping CRC screening than those with longer life expectancy. Despite these positive findings, overall only 43% of patients lowered their intentions to be screened for CRC and only 22% of female patients lowered their intentions to undergo mammography screening. While 78% of PCPs reported using the provided scripts to discuss stopping screening, only 46% reported discussing life expectancy with their older patients and only 22% felt comfortable discussing patient life expectancy. Patients were also mixed about the value of discussing their life expectancy with their PCP, with approximately half (56%) reporting being interested in this information. Primary care providers found the scripts for discussing stopping cancer screening acceptable; however, PCPs may need more intensive training to feel more comfortable discussing patient life expectancy. Reassuringly, none of the patients reported a negative experience discussing stopping screening or their life expectancy with their PCP.

In prior qualitative studies, few older adults report having discussed stopping cancer screening with their PCPs (15,29,30). In this study, 71% of adults older than 75 years reported discussing CRC screening and 75% of females reported discussing mammography screening with their PCP during a routine visit; however, only about half of these patients perceived that their PCPs discussed stopping screening. To be eligible for this study, older adults had to intend to be screened. Older adults who are still enthusiastic about screening and who have received years and years of health messages from clinicians, advocacy groups, friends, family, and the media on the importance of cancer screening may need more than one conversation with their PCP to recognize that their PCP may be offering to stop screening. This may be particularly true for mammography, where screening intentions were even higher than for CRC screening among older women, likely because public health messaging around the importance of mammography screening has been particularly strong and because the harms of a colonoscopy are easier to visualize than the harms of mammography screening. Frameworks on discussing stopping cancer screening have suggested that PCPs may need to introduce and reintroduce this topic (9,31). Future work should aim to observe discussions between PCPs and older adults about stopping screening to learn how to improve these discussions. Also, studies may need to follow patients over time to see if these conversations evolve through more than one encounter.

Although guidelines recommend considering patient life expectancy when deciding whether or not to screen older adults, in qualitative studies older adults often say that they would prefer not to discuss their life expectancy during these discussions (29,32,33). Primary care providers also report discomfort with discussing patient life expectancy in qualitative studies (15,34). This mutual discomfort with discussing patient life expectancy may explain why we found that many PCPs in our study reported using patient life expectancy to inform discussions about stopping cancer screening but often did not directly discuss patient life expectancy. However, 46% of patients thought knowing their life expectancy would be helpful to them when deciding on cancer screening and at least 56% were interested in discussing their life expectancy with their PCP, which is similar to prior studies that have also found that 50%–65% of older adults are interested in discussing their life expectancy with their PCPs, especially as life expectancy decreases (33,35–38). Notably, 78% of patients reported thinking about their life expectancy on their own, suggesting this topic is on the minds of many older adults. Most PCPs also thought it would be important to talk to older adults about their life expectancy but felt uncomfortable with these conversations and felt that they needed more training and practice.

To help PCPs talk to older adults about their long-term prognosis, experts recommend that PCPs assess whether their patients are interested in prognostic information, individualize the content of these discussions, extend the conversation over multiple visits, attend to patient emotion, use visual aids, acknowledge uncertainty, and practice these conversations (28,39–43). This study’s findings support the need to assess patient interest in this information because some patients felt strongly that life expectancy information would be unhelpful to them. This study also found that patients have varying preferences for information on their 10-year life expectancy confirming that these conversations need to be individualized. Because many patients felt that it was impossible to estimate 10-year life expectancy, the study’s findings support the recommendation that PCPs should note the uncertainty in life expectancy estimates. For example, PCPs may acknowledge that prognostic estimates are based on population data and that it is impossible to know any individual person’s future; however, life expectancy estimates from individuals with similar health characteristics may be helpful to the patient in planning for the future and/or in making medical decisions.

The study’s findings suggest that it is acceptable and even helpful to PCPs to provide them with scripts for discussing stopping cancer screening and information on their patients’ 10-year life expectancy. As a next step, investigators will need to consider the broader implementation of this intervention and incorporation into EMRs as recommended by study PCPs. For PCPs interested in currently estimating their patient life expectancy, the Lee–Schonberg index is available on ePrognosis and the scripts are available on request from the author. In addition, the scripts will be used to develop training sessions for PCPs on discussing stopping cancer screening with older adults that will likely include role-playing to allow PCPs to practice using these scripts. Further research is also needed to develop scripts and strategies for PCPs to feel more comfortable bringing up and discussing patient life expectancy. As a next step, investigators will also need to test the effect of this intervention on receipt of cancer screening in older adults in a large randomized clinical trial.

There are limitations to this study. It was a small study of English-speaking patients from one geographic area so findings may not generalize to other regions. Patient refusers had lower educational attainment than participants, but in post-hoc analyses we found no significant differences in patient interest in discussing life expectancy by educational attainment. Primary care providers who chose to complete the questionnaire may be more interested in these discussions; however, 82% of PCPs completed the study questionnaire and nonresponders were similar to responders based on personal characteristics. We provided PCPs with scripts for discussing stopping screening with older adults; however, while we asked PCPs whether they found the scripts helpful, we did not ask them which components of the scripts they used. The study used a quasi-experimental study design; therefore, changes in screening intentions could be due to secular changes. Also, the study examined the intervention’s effect on screening intentions and not the actual screening; however, lower screening intentions have previously been found to be associated with lower screening rates (44).

In summary, providing PCPs with information on their patients’ 10-year life expectancy and scripts for discussing stopping cancer screening was associated with fewer adults older than 75 years intending to undergo CRC screening and fewer females older than 75 years intending to be screened in the next year. While many patients were interested in their 10-year life expectancy and PCPs thought this information would be helpful to patients, PCPs are uncomfortable discussing 10-year life expectancy with older adults. Strategies and training are needed to help PCPs offer and discuss this information. As a next step, investigators need to test the effects of providing PCPs with scripts for discussing stopping cancer screening and information on patient’s 10-year life expectancy on receipt of cancer screening in older adults in a large randomized clinical trial.

## Supplementary Material

igaa027_suppl_Supplementary_Materials
