# Supplementary material for: A Strategy to Prepare Primary Care Clinicians for Discussing Stopping Cancer Screening With Adults Older Than 75 Years
Source: Innov Aging. 2020 Jul 7;4(4):igaa027. doi: 10.1093/geroni/igaa027 (PMC7413618; doi:10.1093/geroni/igaa027)
Supplement: igaa027_suppl_Supplementary_Materials [file igaa027_suppl_supplementary_materials.docx]

**Supplementary Materials Table of Contents:**

**1.** **Supplementary Figure S1:** Venn Diagram of Agreement Between

Different Tools in Estimating Older Adults’ 10-year Life Expectancy Page 2

**2. Supplementary Figure S2:** Study Recruitment Flow for Primary Care Providers Page 3

**3.** **Supplementary Figure S3:** Study Recruitment Flow for Adults 75-89 years Page 4

**4. Supplementary Table S1:** Primary Care Provider (PCP) Participant Characteristics Page 5

**5. Supplementary Table S2.** PCP Characteristics by PCPs refusers and

Non-completers of PCP Survey Page 6

**6. Supplementary Table S3:** Patient Participants vs. Refusers Characteristics Page 7

**7. Supplementary Table S4:** Patient Themes Regarding Discussing Stopping

Cancer Screening and Long-term prognosis/Life expectancy with PCPs Page 8

**8. Supplementary Table S5.** Intentions to be Screened for Cancer

by 10-year Life Expectancy Page 9

**9. Supplementary Table S6:** Themes noted by PCPs in Patient Medical Records

for Screening Decisions Page 11

**10. 10-year mortality risk report example** Page 12

**11. Study questionnaires** Page 14

a. Eligibility

b. Patient Pretest Questionnaire

c. Patient Posttest Questionnaire

d. PCP Questionnaire

e. Chart Abstraction Instrument

**Supplementary Figure S1:** Venn Diagram of Agreement Between Different Tools in Estimating Older Adults’ 10-year Life Expectancy

**2**

**17**

**4**

**67**

a. Schonberg mortality index: Scores ranged from 3 to 23. Mean score: 10.2 (+/-3.8). Scores >10 are associated with >50% chance of 10 year mortality. Thus, adults who score >10 are estimated to have <10 year life expectancy.

b. Lee mortality index: Scores ranged from 4 to 12. Mean score: 7.8 (+/-2.3). Scores >8 are associated with >50% chance of 10 year mortality. Thus, adults who score >8 are estimated to have <10 year life expectancy.

c. Cho et al. estimate life expectancy using U.S. life table data stratified by sex, age (in 5-year age groups), race (White, Black, All), and adjusting for comorbidity. Participant life expectancy ranged from 4.8 to 15.3 years using Cho et al.’s table 3.

**References:**

Cho H, Klabunde CN, Yabroff KR, et al. Comorbidity-adjusted life expectancy: a new tool to inform recommendations for optimal screening strategies. Ann Intern Med 2013; 159(10): 667-76.

Cruz M, Covinsky K, Widera EW, et al. Predicting 10-year mortality for older adults. JAMA 2013; 309(9): 874-6.

Lee SJ, Lindquist K, Segal MR, et al. Development and validation of a prognostic index for 4-year mortality in older adults. JAMA 2006; 295(7): 801-8.

Schonberg MA, Li V, Marcantonio ER, et al. Predicting Mortality up to 14 Years Among Community-Dwelling Adults Aged 65 and Older. J Am Geriatr Soc 2017; 65(6): 1310-5.

**Supplementary Figure S2:** Study Recruitment Flow for Primary Care Providers (PCPs)

5 had patients participate

10 had patients participate

**5 completed PCP questionnaire**

**6 completed PCP questionnaire**, 4 did not

3 had patients participate

Academic Medical Center

68 PCPs

5 Community Practices (1 community health center)

28 PCPs (7 at the community health center)

44 Internal Medicine

PCPS approached

Geriatrics

3 PCPS approached

19 community PCPs approached

5 community health center PCPs approached

40 agreed to have patients participate, 3 refused (1 not a good time, 1 not interested, 1 no reason given), 1 did not respond

3 agreed to have patients participate,

0 refused

10 agreed to have patients participate,

4 refused (2 not interested, 1 not able at this time, 1 no reason given), 5 did not respond

5 agreed to have patients participate, 0 refused

27 had patients participate

**23 completed PCP questionnaire**, 4 did not

**3 completed PCP questionnaire**

PCP Recruitment

**Supplementary Figure S3:** Study Recruitment Flow for Adults 75-89 years

**Excluded or Ineligible (n=2656)**

2211 From review of medical records

389 No CRC screening in the last 10 years

371 Last colonoscopy was abnormal

337 Aged 76-79 with no comorbidity

289 Non-English speaker

221 History of breast cancer

202 Already stopped CRC screening

161 Dementia

78 No mammogram in the last 3 years

68 Already stopped mammography

56 History of colon cancer

12 PCP not participating

10 Last mammogram abnormal

3 Outside of eligible age range,

3 Deceased

3 PCP is a resident

3 Changed PCP

2 Patient has current GI issue

39 PCP did not consent

10 PCP does not want to participate

6 Patient is too sick

4 Patient not appropriate for study

3 Patient is too anxious

2 Already stopped screening

2 Patient has dementia

2 Patient has cancer

2 Patient has serious mental illness

1 Patient is new to PCP

1 Patient does not speak English

1 Patient caring for sick spouse

1 Patient illiterate

4 PCP did not specify

8 During eligibility screen

2 Does not speak English

2 Last colonoscopy abnormal

2 Already stopped mammography screening

1 history of colon cancer

1 Does not have capacity

5 Family declined for patient

358 Research assistant unable to reach patient

35 Research assistant reached patient and patient was interested

but wanted more time to consider but could not be reached

again

**2857** Records assessed for eligibility

**100** Refused or opted-out

29 opted-out of initial telephone contact

71 Patients refused to participate

59 Not interested,

10 Too busy

1 Too sick

1 Bereavement

101 Completed Baseline

10 did not end up seeing their PCP during the study

91 Saw PCP

1 Withdrew

90 Completed Follow-up

**Supplementary Table S1: Primary Care Provider (PCP) Participant Characteristics**

| **PCP Participants** | **Overall (n=37)** |
| --- | --- |
| Race^a^ |  |
| Non-Hispanic White, No. (%) | 28 (76) |
| Non-Hispanic Black, No. (%) | 1 (3) |
| Asian, No. (%) | 4 (11) |
| Hispanic, No. (%) | 4 (11) |
| Site |  |
| Boston academic, No. (%) | 25 (68) |
| Boston community, No. (%) | 12 (32) |
| Age, years |  |
| 20-39, No. (%) | 5 (14) |
| 40-49, No. (%) | 12 (32) |
| 50-59, No. (%) | 11 (30) |
| 60 or older, No. (%) | 9 (24) |
| Female, No. (%) | 20 (54) |
| Role^a^ |  |
| Nurse Practitioner, No. (%) | 1 (3) |
| Internal Medicine physician, No. (%) | 33 (89) |
| Geriatrics physician, No. (%) | 3 (8) |
| Proportion of patients panel 75+ years |  |
| <10%, No. (%) | 3 (8) |
| 10-<20%, No. (%) | 11 (30) |
| 20-<30%, No. (%) | 11 (30) |
| 30% or more | 12 (32) |
| Years in role^a^ |  |
| <5 years, No. (%) | 2 (5) |
| 5-<15 years, No. (%) | 9 (24) |
| 16-25 years, No. (%) | 12 (32) |
| >25 years, No. (%) | 14 (38) |
| Number of patients seen per week^a^ |  |
| 30 or less, No. (%) | 15 (41) |
| 31 to 60, No. (%) | 15 (41) |
| >60, No. (%) | 7 (19) |
| Average time for follow-up visits^a^ |  |
| 15 minutes, No. (%) | 4 (11) |
| 20 minutes, No. (%) | 18 (49) |
| 25 minutes, No. (%) | 8 (22) |
| 30 minutes, No. (%) | 7 (19) |
| No. of patients participated^a^, mean (SD) | 2.0 (1.2) |
| 1, No. (%) | 16 (43) |
| 2-3, No. (%) | 18 (49) |
| >3, No. (%) | 3 (8) |

a. Proportions do not add to 100% due to rounding.

**Supplementary Table S2:** PCP Characteristics by PCPs Refusers and Non-completers of PCP Survey

| **Characteristic** | **PCPs approached to participate (n=65)** | **PCPs who agreed to participate (n=58)** | **PCPs who had participating patients (n=45)** | **PCPs who completed survey (n=37)** |
| --- | --- | --- | --- | --- |
| Race |  |  |  |  |
| Non-Hispanic White, No. (%) | 51 (78.5) | 47 (81.0) | 35 (77.8) | 28 (75.7) |
| Non-Hispanic Black, No. (%) | 1 (1.5) | 1 (1.7) | 1 (2.2) | 1 (2.7) |
| Asian, No. (%) | 9 (13.9) | 6 (10.3) | 5 (11.1) | 4 (10.8) |
| Hispanic, No. (%) | 4 (6.2) | 4 (6.9) | 4 (8.9) | 4 (10.8) |
| Site |  |  |  |  |
| BIDMC academic | 45 (69.2) | 42 (72.4) | 29 (64.4) | 25 (67.6) |
| BIDMC community | 20 (30.8) | 16 (27.6) | 16 (35.6) | 12 (32.4) |
| Female | 36 (55.4) | 33 (56.9) | 23 (51.1) | 20 (54.0) |
| Role |  |  |  |  |
| Nurse Practitioner | 1 (1.5) | 1 (1.7) | 1 (2.2) | 1 (2.7) |
| Internal Medicine physician | 59 (90.8) | 53 (91.4) | 40 (88.9) | 33 (89.2) |
| Geriatrics physician | 4 (6.2) | 3 (5.2) | 3 (6.7) | 3 (8.1) |
| Family Medicine physician | 1 (1.5) | 1 (1.7) | 1 (2.2) | 0 |

**Supplementary Table S3:** Patient Participants vs. Refusers characteristics

| **Characteristic** | **Participants (n=90)** | **Refusers (n=100)** | **p-value** |
| --- | --- | --- | --- |
| Age, mean (SD), y | 80.0 (2.9) | 80.7 (3.7) |  |
| Age Group |  |  |  |
| 76-79, No. (%) | 46 (51) | 64 (64) |  |
| 80-84, No. (%) | 37 (41) | 22 (22) |  |
| 85-89, No. (%) | 7 (8) | 14 (14) |  |
| Gender |  |  | 0.28 |
| Male | 47 (52.2) | 60 (60.0) |  |
| Female | 43 (47.8) | 40 (40.0) |  |
| Race |  |  | 0.59 |
| Non-Hispanic White, No. (%) | 78 (88) | 80 (80) |  |
| Non-Hispanic Black, No. (%) | 8 (9) | 15 (15) |  |
| Hispanic, No. (%) | 1 (1) | 2 (2) |  |
| Other, No. (%) | 2 (2) | 3 (3) |  |
| Recruitment site |  |  | 0.56 |
| BIDMC academic, No. (%) | 53 (59) | 63 (63) |  |
| BIDMC community, No. (%) | 37 (41) | 37 (37) |  |
| Education |  |  | <0.0001 |
| <High school, No. (%) | 4 (4) | 7 (7) |  |
| High school, No. (%) | 11 (12) | 29 (29) |  |
| Some college, No. (%) | 16 (18) | 5 (5) |  |
| College degree or beyond, No. (%) | 59 (66) | 55 (55) |  |
| Missing, No. (%) | 0 | 4 (4) |  |

**Supplementary Table S4:** Intentions to be Screened for Cancer by 10-year Life Expectancy.^a^

| **Outcomes** | **Baseline**  **n=90** | **Follow-up**  **n=90** | **p-value** |
| --- | --- | --- | --- |
| **Colorectal Cancer Screening (CRC)** |  |  |  |
| Intentions to be screened with colonoscopy^b^, |  |  |  |
| <10 year life expectancy, mean (SD) | 7.2 (5.4) | 3.9 (5.4) | 0.21^c^ |
| >10 year life expectancy, mean (SD) | 10.5 (4.9) | 8.7 (5.9) |  |
| Missing, no | 0 | 2 |  |
| Any change in intentions: |  |  |  |
| <10 year life expectancy |  |  |  |
| Intentions moved towards CRC screening, No. (%) |  | 4 (9) | 0.60^c^ |
| Intentions stayed the same, No. (%) |  | 19 (40) |  |
| Intentions moved away from CRC screening, No. (%) |  | 22 (47) |  |
| Missing |  | 2 |  |
| >10 year life expectancy |  |  |  |
| Intentions moved towards CRC screening, No. (%) |  | 6 (14) |  |
| Intentions stayed the same, No. (%) |  | 20 (46) |  |
| Intentions moved away from CRC screening, No. (%) |  | 17 (40) |  |
| **Mammography Screening (women only)** | n=43 | n=43 |  |
| Intentions to be screened with mammography^d^ |  |  | 0.56^c^ |
| <10 year life expectancy, mean (SD) | 12.6 (3.9) | 11.0 (5.3) |  |
| >10 year life expectancy, mean (SD) | 13.1 (2.5) | 12.1 (4.7) |  |
| Missing, No. | 0 | 2 |  |
| Any change in intentions |  |  |  |
| <10 year life expectancy |  |  | 0.51^c^ |
| Intentions moved towards mammography, No. (%) |  | 1 (7) |  |
| Intentions stayed the same, No. (%) |  | 9 (60) |  |
| Intentions moved away from mammography, No. (%) |  | 4 (27) |  |
| Missing |  | 1 (7) |  |
| >10 year life expectancy |  |  |  |
| Intentions moved towards mammography, No. (%) |  | 1 (4) |  |
| Intentions stayed the same, No. (%) |  | 21 (75) |  |
| Intentions moved away from mammography, No. (%) |  | 5 (18) |  |
| Missing, No. |  | 1. (4) |  |

a. 10-year life expectancy was determined using the Lee-Schonberg index (whichever gave the lower prognosis)

b. Intentions to be screened for colorectal cancer- 1(will not have a colonoscopy/stool card in the next few years) to 8 (undecided) to 15 (I will have a colonoscopy/stool care in the next few years)

c. The p value is the p value for the effect by life expectancy.

d. Intentions to be screened for breast cancer with mammography- 1(I will not have a mammogram in the next year) to 8 (undecided) to 15 (I will have a mammogram in the next year)

**Supplementary Table S5:** Patient Themes Regarding Discussing Stopping Cancer Screening and Long-Term Prognosis/Life expectancy with PCPs.^a^

| **Patient Themes** | **Example Quotes** |
| --- | --- |
| **Enthusiasm for screening** | “I see no reason not to do it.” “I will do whatever I can do to stop myself from getting cancer.” |
| Family/friend had late life cancer and did well | “I knew someone that had a mammogram at 91 and they found cancer. She went through treatment and is still alive and all right.” |
| Concerns about ageism | “Because I’m turning 80 doesn’t mean I give up on preventive health issues. There is so much ageism.” “Everyone is living longer. I don’t want people to give up on me.” |
| Family history | “I do have a family history so my doctor recommends a colonoscopy every 5 years.” |
| In good health | “I think they should give you screening as long as you appear healthy.” |
| Enthusiasm for mammography > colonoscopy | “I am more interested in mammograms. I already decided I was done with colonoscopies.” |
| **Patients want value** | “If she could show me that not having one was better than having one I would trust that.” |
| **Weaning** | “We decided to skip this year and revisit at a later time.” |
| **Stopping screening novel** | “Honestly it is the first time any doctor had talked to me about this.” |
| **Differing preferences for discussing life expectancy** | “It is a personal choice people want to know or don't” “I could talk to her about it.”  “I don't really want to talk about that with my doctor.” |
| Helpful | “I think talking about death is always helpful.” “I think it would be valuable to people like me who are inquisitive and interested in this.” |
| Not helpful | “I see no utility in discussing that.” |
| Depends on the patient | “I think it is a good idea if the patient is agreeable and receptive to those discussions.” |
| Only if sick or life expectancy short | “If I’m in bad health. I might want to know how long.”  “As long as I'm in good health, I don't think there is any need for a conversation of this nature.” |
| Impossible to predict | “Until somebody develops a crystal ball that I believe in I don't want to know.”  “It's just a guess and there is no validity to it.” |
| **Discomfort with specific life expectancy estimates** | “I can’t even wrap my head around the fact that someone could give me a number like this.”  “I don't think you want to tell people exact numbers it could cause depression.”  “I don’t know if I want anybody to say I’ve only got 5 years or 10 years.” |
| **Estimates are valuable** | “I think they (prognosis/life expectancy estimates) are all valuable.” |
| **Already receives estimates from insurance company** | “My life insurance company did an algorithm and it said i would probably live for another 17 years.” “Life insurance companies probably have a better sense of longevity with their metrics.” |
| **Talks to others about life expectancy** | “I discuss it with many other people so I didn't think I had to discuss it with my doctor.”  “I've discussed this with my wife and children.” |
| **PCPs need training** | “If the healthcare industry is going to go down this road they need to do extensive training for doctors on how to do this. I don’t think the average PCP has a clue.” |
| **Patients estimate their own life expectancy** | “It’s not like this topic isn’t something I have thought about on my own.”  “I think about it frequently.” |
| **Patients reluctant to bring up life expectancy with PCP** | “I never would have said anything but I had been wondering how things are going.”  “It would be good for a PCP to say would you like to talk about how long you have to live.” |

a. Codes were grouped into major themes which are highlighted in bold

**Supplementary Table S6:** Themes Noted by PCPs in Patient Medical Records for Screening Decisions^a^

| **Factors influencing screening decisions** | **Example Quotes** |
| --- | --- |
| **Age** | She has aged out of screening |
| **Family history** | “Very strong family history of breast cancer in mother, sister, and aunt.”  “She has a family history of colon cancer and so she would like to continue with screening.” |
| **Good health** | “Given that he had an adenoma and he still is quite healthy, it seems reasonable to do this again.” “Her estimated life expectancy was calculated to be 11.4 years, with a 63% chance of living > 10 years. She elected to continue with both mammograms and colonoscopies.” |
| **Patient preference** | She wants to continue with colon cancer screening and breast cancer screening. |
| **Up to date, therefore defer decision to stop** | “He is not yet due for colon cancer screening. Thus, we will defer further decisions.”  “She will decide about colon cancer screening when it is 10 years from her last colonoscopy.” |
| **Cancer screening scheduled** | He is scheduled for colonoscopy next week. |
| **History of Polyps** | “Given her history of polyps and poor prep, a repeat colonoscopy in 3 years was recommended.” “She once had an abnormal, but subsequent colonoscopies have only showed hyperplastic polyps and she prefers not to have further.” |
| **Lack of trial data** | “Given her age and lack of supporting data, patient is agreeable to discontinue screening.” |
| **Risks outweigh benefits** | “We discussed the pros and cons of continuing mammograms at her age, reviewed risk of missing a breast cancer vs. benefit of avoiding harms from overdiagnosing or overtreating. She is comfortable discontinuing mammograms.” |
| **Wean first** | “She would like to have one more mammogram at the age of 80.”  “We agreed to do every other year screening and will defer this year.” |
| **Inadequate past screening** | “We have discussed several times repeating this [colonoscopy] given the poor prep.” |

a. Codes were grouped into major themes which are highlighted in bold.

**Example Risk Report:**

**Patient prognosis**: *Your patient has 60% chance of dying in 10 years; her estimated life expectancy is <10 years.*

*This estimate is from the Lee-Schonberg mortality indices that use the information in the table below. Adults* with >50% chance of dying in 10 years are estimated to have <10 year life expectancy

| Risk Factor Information (Lee/Schonberg Index^12^) |
| --- |

|  |  |  |  |  |  |
| --- | --- | --- | --- | --- | --- |
| Age | 75-79 | 80-84 | 85+ |  |  |
| Sex | Female | Male |  |  |  |
| Body mass index | <25 | 25 or more |  |  |  |
| Patient says their health is | Excellent | Very Good | Good | Fair | Poor |
| Cigarette use | Never | Former | Current |  |  |
| Number of hospitalizations in the past year | None | 1 time | 2 or more times |  |  |
| History of cancer (excluding minor skin cancers) | yes | no |  |  |  |
| Emphysema/COPD | yes | no |  |  |  |
| Oxygen dependent | yes | no |  |  |  |
| Diabetes (including borderline/pre-diabetes) | yes | no |  |  |  |
| Congestive Heart Failure | yes | no |  |  |  |
| Patient needs help from other people with household chores or shopping because of a physical, mental, or emotional problem | yes | no |  |  |  |
| Difficulty walking a quarter mile (by themselves without using any special equipment | yes | no |  |  |  |
| Difficulty managing finances on their own | yes | no |  |  |  |
| Difficulty pulling or pushing large objects such as a living room chair | yes | no |  |  |  |
| Difficulty with bathing or showering without help from other people | yes | no |  |  |  |

**Table 3 by Cho et al.^1^ can be used to get a quick estimate of patient life expectancy (based on patient age, sex, race, and comorbidities).**

**SEE CHO et al**

**Because your patient is a white woman aged 88 with a history of melanoma her estimated life expectancy is 6.6 years. Comorbidity burden was determined using the Charlson Comoribidty Index.**

**References:** 1. Cho H, Klabunde CN, Yabroff KR, Wang Z, Meekins A, Lansdorp-Vogelaar I, Mariotto AB. Comorbidity-adjusted life expectancy: a new tool to inform recommendations for optimal screening strategies. Ann Intern Med, 2013. 159(10): p. 667-76.

2. Schonberg MA, Davis RB, McCarthy EP, Marcantonio ER. Index to predict 5-year mortality of community-dwelling adults aged 65 and older using data from the National Health Interview Survey. J Gen Intern Med, 2009. 24(10): 1115-22.2.

3. [Lee SJ, Lindquist K, Segal MR, Covinsky KE. Development and validation of a prognostic index for 4-year mortality in older adults. JAMA. 2006 Feb 15;295(7):801-8.](http://www.ncbi.nlm.nih.gov/pubmed/16478903)

4. Charlson ME, Pompei P, Ales KL, MacKenzie CR. A new method of classifying prognostic comorbidity in longitudinal studies: development and validation. J Chronic Dis, 1987. 40(5): p. 373-83.

**Eligibility-Capacity**

*Before I continue, I would like to confirm that you are eligible for this study:*

1. For women only: Have you ever been diagnosed with breast cancer (including in situ or non-invasive breast cancer)?

□_0_ No

□_1_ Yes (ineligible)

For all:

2. Have you ever been diagnosed with colon cancer?

□_0_ No

□_1_ Yes (ineligible)

3. Are you in hospice care?

□_0_ No □_1_ Yes (ineligible)

Now, I am going to ask you whether you plan to get screened for cancer.

4. From 1-15, how likely are you to get a colonoscopy or stool cards to look for colon cancer in the future, 1 means you are certain you will get a colonoscopy or complete stool cards in the next few years and 15 means you do not plan to get a colonoscopy or stool cards in the next few years. If you are not sure, please give a number between 1 and 15 that best describes how likely you are to get a colonoscopy or stool cards; 8 is the middle

In the next few years:

1 2 3 4 5 6 7 8 9 10 11 12 13 14 15

I Will NOT I am Undecided I Will Get a

Get a colonoscopy/stool cards colonoscopy/stool cards

Women:

5. Similarly, from 1-15, how likely are you to get a mammogram in the next 2 years, 1 means you are certain you will get a mammogram and 15 means you do not plan to get a mammogram in the next 2 years. If you are not sure, please give a number between 1 and 15 that best describes how likely you are to get a mammogram; 8 is the middle

In the next 2 years:

1 2 3 4 5 6 7 8 9 10 11 12 13 14 15

I Will NOT I am Undecided I Will Get a

Get a Mammogram Mammogram

(If a man answers between 11-15 on colonoscopy then he is not eligible. If a woman answers between 11-15 on both mammography and colonoscopy then she is not eligible)

If ineligible: Thank you for speaking with me on the phone. Based on your answers to the questions above this study is not appropriate for you. Thank you again for your time and patience and willingness to hear about the study.

6. Memory test. **Orientation-Memory-Concentration Test Short Blessed Test (SDT)**

| **Question** | **Maximum Error** | **Score X** | **Weight** |
| --- | --- | --- | --- |
| What year is it now? | 1 | ____ x 4 | = ____ |
| What month is it now? Repeat this phrase and I will ask you to remember it and tell it to me later: John Brown, 42 Market Street, Chicago | 1 | ____ x 3 | = ____ |
| About what time is it? (within one hour) | 1 | ____ x 3 | = ____ |
| Count backwards 20 to 1 | 2 | ____ x 2 | = ____ |
| Say the months in reverse order | 2 | ____ x 2 | = ____ |
| Repeat the phrase just given | 5 | ____ x 2 | = ____ |
| **Total error score = _____** | | | |

**Score 10 or more then ineligible.**

**If ineligible: Thank you for speaking with me today. Your answers to some of the questions above suggest that the study may cause you burden. Therefore, this study is not appropriate for you and I will stop the interview now. Thank you for your time. I greatly appreciate your kindness.**

**Comments:_**

**CAPACITY FOR INFORMED CONSENT**

Interviewer ID#:___ ___ Date __ __/__ __/__ __ __ __

[Ask the patient the questions in **bold** and rate the statement below each question based on patients’ answer]

**Now, we would just like to verify your understanding of the study. This is a part of our standard procedure**.

| *Ability to understand that participation is a choice:*  **1. Can you decide not to participate in this study?** |  | |
| --- | --- | --- |
| Patient understands that participation in the study is completely voluntary. | Correct | Incorrect |
| **2. Can you quit the study after you have agreed to participate?**  Patient is aware that he/she can discontinue study participation at any time. | Correct | Incorrect |
| *Ability to understand relevant information:* |  |  |
| **3. Can you please describe in your own words what this study is about?**  Patient can describe the purpose of the study. | Correct | Incorrect |
| **4. What are the main risks of participation in this study?**  Patient understands that there are some small risks associated with the study  (e.g. loss of privacy). | Correct | Incorrect |
| **5. What are the potential benefits of participation in this study?**  Patient understands that he/she will have an opportunity to learn about preventive measures for older women.  abaaboumore about health promotion and that the study will benefit future patients. | Correct | Incorrect |
|  |  |  |
| *Ability to appreciate the situation and its likely consequences*  **6. Choosing not to participate in this study will not affect your medical care. (Is this statement correct or incorrect?)**  Patient understands that declining participation will not affect medical care. | Correct | Incorrect |

**7. Will the information you give us for this study be kept secret and confidential within the study personnel and its affiliates?**

Patient understands that all their information will be kept confidential. Correct Incorrect

***Overall capacity to provide informed consent:***

Total # of “Incorrect”: /7 If “Incorrect” for 3 or more questions, patient **cannot** be enrolled

***If you are uncertain, continue with the baseline AND contact study director.***

**If ineligible: Thank you for speaking with me today. Your answers to some of the questions above suggest that the study may cause you burden. Therefore, this study is not appropriate for you and I will stop the interview now. Thank you for your time. I greatly appreciate your kindness.**

**Comments:_**

**Patient Pretest Questionnaire**

*Thank you for agreeing to participate in this study.*

Date:___________________________ Time to complete:_____________________

*I would like to gather a little more background information about you and your health:*

*(Questions needed for prognostic indices)*

1. How old are you? ____________________________

2. Would you say your health in general is:

□_0_ Excellent

□_1_ Very Good

□_2_ Good

□_3_ Fair

□_4_ Poor

3. Because of a physical, mental or emotional problem, do you need help from other people with routine needs? These include everyday household chores, shopping or getting around for other purposes.

□_0_ No

□_1_ Yes

4. Because of a health or memory problem do you have any difficulty with managing your money-such as paying your bills and keeping track of expenses?

□_0_ No

□_1_ Yes

5. Because of a health or memory problem do you have any difficulty with bathing or showering?

□_0_ No

□_1_ Yes

6. Because of a health problem do you have any difficulty with pulling or pushing large objects such as a living room chair?

□_0_ No

□_1_ Yes

7. By yourself and without using any special equipment, how difficult is it for you to walk a quarter of a mile (about 3 city blocks)?

□_0_ Not at all difficult

□_1_ A little difficult

□_2_ Very difficult

□_3_ Can’t do at all/do not do/can only do with a cane or walker

8. Which best describes your cigarette use?

□_0_ Never smoked or smoked less than 100 cigarettes in your life

□_1_ Former smoker

□_3_ Current smoker

9. During the past 12 months, how many times were you hospitalized overnight?

□_0_ None □_1_ Once □_3_ Twice or more

10. How much do you weigh? __________

11. How tall are you?_____ feet inches________

**Adult Functional Assessment**

**Activities of Daily Living and Instrumental Activities of Daily Living**

***The next set of questions is about your ability to perform daily activities. I'm going to describe an activity, and I'd like you tell me whether you can perform this activity with no help, with some help, or with a lot of help.***

| IADLs | Needs no help | Needs some help | Needs a lot of help | Refused | Don’t know |
| --- | --- | --- | --- | --- | --- |
| 12. Cooking: planning, preparing, cooking full meals | 2 | 1 | 0 | 98 | 99 |
| 13. Walking: in and around home, even and uneven surfaces, steps, cane/walker or other aid OK | 2 | 1 | 0 | 98 | 99 |
| 14. Home chores: make bed, sweep/vacuum, laundry, moving a chair | 2 | 1 | 0 | 98 | 99 |
| 15. Transportation: getting places via car, cab, bus, subway, rides | 2 | 1 | 0 | 98 | 99 |
| 16. Shopping: for groceries, clothes, necessities | 2 | 1 | 0 | 98 | 99 |
| 17. Using telephone: finding and dialing number | 2 | 1 | 0 | 98 | 99 |
| 18. Managing money: paying bills, writing checks | 2 | 1 | 0 | 98 | 99 |
| 19. Taking medications: proper type, dose, time | 2 | 1 | 0 | 98 | 99 |
| ADLs | Needs no help | Needs some help | Completely unable | Refused | Don’t  know |
| 20. (Bathing) Going to and from shower/tub | 2 | 1 | 0 | 98 | 99 |
| 21. (Dressing) Picking out clothes, buttoning, zipping, tying | 2 | 1 | 0 | 98 | 99 |
| 22. (Toileting) Going to and from toilet on time | 2 | 1 | 0 | 98 | 99 |
| 23. (Transferring) Getting up and down from bed and chair | 2 | 1 | 0 | 98 | 99 |
| 24. (Feeding) Eating, cutting your food, using utensils, drinking | 2 | 1 | 0 | 98 | 99 |

**COMORBIDITY - Now I will ask you more questions about your medical history.**

|  | No | Yes | Maybe | Refused | DK |
| --- | --- | --- | --- | --- | --- |
| 25. Have you ever been told by a doctor or health professional that you had a heart attack (also called myocardial infarction)? | 0 | 1 | 2 | 98 | 99 |
| 26. Have you ever been told by a doctor or health professional that you have heart failure? | 0 | 1 | 2 | 98 | 99 |
| 27. Have you had an operation to unclog or bypass arteries in your legs? (peripheral vascular disease) | 0 | 1 | 2 | 98 | 99 |
| 28. Have you ever had a stroke or transient ischemic attack (TIA)? | 0 | 1 | 2 | 98 | 99 |
| 29. Do you have difficulty moving an arm or leg as a result of the stroke? | 0 | 1 | 2 | 98 | 99 |
| 30. Do you have asthma? | 0 | 1 | 2 | 98 | 99 |
| 31. Have you ever been told by a doctor or health professional that you have emphysema or COPD? [if answer “no”, skip to #**42**] | 0 | 1 | 2 | 98 | 99 |
| 32. If yes, do you use oxygen at home? | 0 | 2 |  | 98 | 99 |
| 33. Do you have stomach ulcers, or peptic ulcer disease? | 0 | 1 | 2 | 98 | 99 |
| 34. Have you ever been told by a doctor or health professional that you have diabetes (high blood sugar)? | 0 | 1 | 2 | 98 | 99 |
| 35. Have you ever had poor kidney function (blood test showing high creatinine)? | 0 | 1 | 2 | 98 | 99 |
| 36. Have you ever needed hemodialysis or peritoneal dialysis? | 0 | 1 | 2 | 98 | 99 |
| 37. Do you have **rheumatoid** arthritis? | 0 | 1 | 2 | 98 | 99 |
| 38. Do you have Lupus (systemic lupus erythematosus)? | 0 | 1 | 2 | 98 | 99 |
| 39. Do you have polymyalgia rheumatica? | 0 | 1 | 2 | 98 | 99 |
| 40. Do you have cirrhosis or serious liver damage? (for example you have abnormal liver tests) | 0 | 1 | 2 | 98 | 99 |
| 41. Have you had leukemia? | 0 | 1 | 2 | 98 | 99 |
| 42. Have you had lymphoma? | 0 | 1 | 2 | 98 | 99 |
| 43. Have you ever been told by a doctor or health professional that you have cancer [exclude minor skin cancers]? | 0 | 1 | 2 | 98 | 99 |

44. All in all, you have complete trust in your doctor:

1 = strongly disagree

2 = somewhat disagree

3 = neutral

4 = somewhat agree

5 = strongly agree

45. How would you define your marital status?

□ Single (never married) ^0^

□ Currently married or living as married ^1^

□ Divorced ^2^

□ Separated ^3^

□ Widowed ^4^

□ Don’t know ^99^

46. Do you live alone or with others?

□ I live alone ^0^

□ I live with others ^1^

47. What is the highest level of school you have completed or the highest degree you have received?

□ <6^th^ grade^0^
 □ 6^th^ grade^1^

□ 7^th^ grade^2^

□ 8^th^ grade

□ 9^th^ grade

□ 10^th^ grade

□ 11^th^ grade

□ GED or equivalent^3^

□ High school or less, no diploma^4^

□ High school graduate^5^

□ Some college or an Associate’s degree^6^

□ Bachelor’s degree (e.g., BA, AB, BS, BBA)^7^

□ Master’s degree (e.g., MA, MS, MEngineering, MEducation, MBA)^8^

□ Professional School degree (e.g, MD, DDS, DVM, JD)^9^

□ Nursing degree^10^

□ Doctoral degree (e.g., PhD, EdD, ScD)^11^

48. How confident are you filling out medical forms by yourself?

□_0_ Extremely confident

□_1_ Quite a bit confident

□_2_ Somewhat confident

□_3_ A little confident

□_4_ Not at all confident

49. How often do you have someone like a family member, friend, hospital, or clinic worker or

caregiver, help you read hospital materials?

□_0_ always

□_1_ often

□_2_ sometimes

□_3_ rarely

□_4_ Never

50. How often do you have problems learning about your medical condition because of difficulty understanding written information?

□_0_ always

□_1_ often

□_2_ sometimes

□_3_ rarely

□_4_ Never

51. Do you consider yourself to be Hispanic or Latino?

□ No, I am not Hispanic or Latino ^0^

□ Yes, I am Hispanic or Latino ^1^

□ Don’t know ^99^

52. Which of the following racial groups do you most identify with?

□ White or Caucasian ^0^

□ Black or African American ^1^

□ Asian ^2^

□ American Indian or Alaska Native ^3^

□ Native Hawaiian or other Pacific Islander ^4^

□ Other ^5^

53. Of these income groups can you tell me which best represents your total combined household income during the last year?

□ <$20,000 ^0^

□ $20,000-$35,000 ^1^

□ $36,000-$65,000 ^2^

□ $66,000-$100,000 ^3^

□ >$100,000 ^4^

Prefer not to answer

□ Don’t know ^99^

**Additional background questions for Aim 2:**

For women only:

54. Do you have a family history of breast cancer in a mother, sister, or daughter?

□_0_ No (skip to 30)

□_1_ Yes

55. Did your mother have breast cancer?

□_0_ No

□_1_ Yes

56. Did your sister have breast cancer: (if so, how many sisters had breast cancer?)

0 1 2 3 4 ___

57. Did your daughter have breast cancer: (if so, how many daughters had breast cancer?)

0 1 2 3 4 ___

58. Have you ever had a breast biopsy?

□_0_ No (Skip to 6)

□_1_ Yes, I have had one breast biopsy

□_2_ Yes, I have had two or more breast biopsies

58b. If yes to 58, What did your breast biopsy find:

Atypical hyperplasia

Fibroadenoma

Other

I don’t know, it was negative

For all participants:

59. Do you have a family history of colon cancer in a biological parent, sibling, or child?

□_0_ No (skip to 30)

□_1_ Yes

60. Did your parent have colon cancer?

□_0_ No

□_1_ Yes

61. Did your sibling have colon cancer: (if so, how many siblings had colon cancer?)

0 1 2 3 4 ___

62. Did your child have colon cancer: (if so, how many children had colon cancer?)

0 1 2 3 4 ___

63. When do you plan to get your next mammogram?

□_0_ I do not plan on getting another mammogram

□_1_ In the next year

□_2_ More than 1 year from now but less than 2 years from now

□_3_ More than 2 years from now

□_4_ More than 3 years from now but less than 5 years from now

□_5_ Other:____________________

64. Some doctors are beginning to talk to adults over age 75 about how long they may have to live to help them plan for the future, are you interested in talking to your doctor about how long you may have to live?

Not at all

A little

Somewhat

A great deal

65. Why/why not: (write down what patient says)

**Thank you for completing the baseline questionnaire.**

Please let me know if I can answer any questions. My phone number is ________________ if you need to reach me. My contact information is also included in the letter that you received about this study.

**Patient Post-test Questionnaire**

**Study ID: ___________**

**Date:_______________**

*Thank you for agreeing to participate in this study. We are interested in learning about your visit with your primary care doctor and/or nurse practitioner (NP) and about your thoughts about cancer screening.*

For Women:

**Intentions:**

1. First, From 1-15, how likely are you to get a mammogram in the next year, 1 means you are certain you will get a mammogram and 15 means you do not plan to get a mammogram in the next year. If you are not sure, please give a number between 1 and 15 that best describes how likely you are to get a mammogram; 8 is the middle

In the next year:

1 2 3 4 5 6 7 8 9 10 11 12 13 14 15

I Will I am Undecided I Will **NOT** Get a Get a Mammogram

Mammogram

2. When do you plan to get your next mammogram?

□_0_ I do not plan on getting another mammogram

□_1_ I plan to get one more mammogram in the next year and then stop

□_2_ I plan to get one more mammogram in the next 2 years and then stop

□_3_ I plan to get another mammogram more than 2 years from now

□_5_ Other:____________________

3. At today’s (or this week’s) visit, did your primary care doctor or nurse practitioner talk to you about stopping to go for mammograms?

□_1_  YES □_0_  NO

4. If no, has your primary care doctor or nurse practitioner talked to you about stopping to go for mammograms in the past?

□_1_  YES □_0_  NO

5. Has your doctor talked to you about the downsides to getting a mammogram?

□_1_  YES □_0_  NO

6. What does your primary care doctor (or nurse practitioner) recommend in terms of mammograms? (please select the best option)

□_0_ That you continue getting mammograms

□_1_ That you get another mammogram but stop after the next one

□_2_ That you stop getting mammograms for now but reconsider later

□_3_ That you stop getting mammograms

□_4_ Made no recommendation and told me it is my choice

□_5_ We did not discuss mammograms

□_6_ Other:________________

For all participants:

**Intentions:**

7. From 1-15, how likely are you to get a colonoscopy or stool cards to look for colon cancer, 1 means you are certain you will get a colonoscopy in the next few years (or do stool cards in the next year) and 15 means you do not plan to get a colonoscopy or do stool cards in the future. If you are not sure, please give a number between 1 and 15 that best describes how likely you are to get a colonoscopy or do stool cards; 8 is the middle

In the next few years:

1 2 3 4 5 6 7 8 9 10 11 12 13 14 15

I Will I am Undecided I Will NOT Get a

Get a colonoscopy/stool cards Colonoscopy/stool cards

8. *If patient still plans to get colon cancer screening ask:* Do you plan to get:
 Stool cards

Colonoscopy

Other:_____________

9. At today’s (or this week’s) visit, did you talk to your primary care doctor or nurse practitioner about when you would stop getting colonoscopies or stool tests to look for colon cancer?

□_1_  YES □_0_  NO

10. If no, has your primary care doctor or nurse practitioner talked to you about when you would stop getting colonoscopies or stool tests to look for colon cancer?

□_1_  YES □_0_  NO

11. Has your doctor talked to you about the downsides to getting a colonoscopy?

□_1_  YES □_0_  NO

12. Has your doctor talked to you about the downsides to getting stool cards?

□_1_  YES □_0_  NO

13. What does your primary care doctor or nurse practitioner recommend? (please select the best

option)

□_0_ That you continue having colonoscopies or stool tests

□_1_ That you get another colonoscopy or stool test but stop after that

□_2_ To stop getting colonoscopies or stool tests

□_3_ Made no recommendation and told me it is my choice

□_4_ We did not discuss colonoscopies or stool tests

□_5_ Other:________________

Most cancers found on an older adult’s mammogram (women) or a colonoscopy (all) are slow growing such that it usually takes at least 10 years before a patient has a chance of benefiting from getting a mammogram (women) or a colonscopy (all). A person that gets a mammogram or colonoscopy now may avoid death from breast or colon cancer but not for 10 years. Therefore, doctors are being encouraged to talk to older adults about how long they may have to live so that patients can think about whether it makes sense for them to keep getting mammograms or colonoscopies.

14. Have you ever thought about how long you may have to live?

□_1_  YES □_0_  NO □_2_  I am not sure

15. Did your primary care doctor or nurse practitioner talk to you today about how long you may have to live?

□_1_  YES □_0_  NO □_2_  I am not sure

16. *If no,* has your primary care doctor or nurse practitioner talked to you in the past about how long you may have to live?

□_1_  YES □_0_  NO □_2_  I am not sure

17. Are you interested in talking to your doctor about how long you may have to live to help plan for your future?

Not at all

A little

Somewhat

A great deal

I don’t know

Why/why not: (write down what patient says)

18. *If yes to having had a discussion now or in the past,* did your primary care doctor give you an estimate for how long you may have to live?

□_1_  YES □_0_  NO □_2_  I am not sure

19. How did your primary care doctor give you information about how long you may have to live?

(Write down what patient says)

20. How would you like for your doctor to give you information about how long you may have to live?
(please rank in terms of preference)

As a range, for example “5-10 years”

As a probability, for example “50/50 chance of living 10 years”

As a number, for example “10 years”

Other

21. Did your primary care doctor ask you about you thoughts about how long you may have to live?

□_1_  YES □_0_  NO □_2_  I am not sure

22. *If patients had a discussion,* how helpful was it to talk to your primary care doctor about how long you may have to live?

4 very helpful

3 somewhat helpful

2 a little helpful

1 not helpful

0 unhelpful

23. *If patients had a discussion,* talking to your primary care doctor about how long you may have to live made you feel:

0 not anxious at all

1 a little anxious

2 somewhat anxious

3 very anxious

4 extremely anxious

24. Has another doctor or health care professional talked to you about how long you may have to live (e.g., a surgeon, a specialist)?

_1_  YES □_0_  NO □_2_  I am not sure

25. If yes,

What kind of doctor was it?

Surgeon

Cardiologist

Gastroenterologist

Rheumatologist

Pain specialist

Psychologist

Psychiatrist

Nephrologist

Neurologist

Other:

26. How did this doctor give you information about how long you may have to live?

(Write down what patient says)

27. How helpful was it to talk to this doctor about how long you may have to live?

4 very helpful

3 somewhat helpful

2 a little helpful

1 not helpful

0 unhelpful

28. Talking to this doctor about how long you may have to live made you feel:

0 not anxious at all

1 a little anxious

2 somewhat anxious

3 very anxious

4 extremely anxious

29. Has another professional (e.g., a financial planner, clergy) talked to you about how long you may have to live?

□_1_  YES □_0_  NO □_2_  I am not sure

30. If yes, which professional (check all applicable):

1. Insurer

2. financial planner

3. clergy

4. other:_____________________

31. Have your children or another family member/friend talked to you about how long you may have to live?

□_1_  YES □_0_  NO □_2_  I am not sure

32. If yes, which family member or friend:

1. daughter

2. son

3. niece

4. nephew

5. spouse

6. other

**Factors in Talking to Primary care doctor about how long you may have to live**

***These next questions are about factors that* may influence you about *whether or not to talk to your primary care doctor about how long you may have to live. Would each of the following factors influence you? Please answer yes or no.***

| Read:  33. Would the following factors influence you in terms of wanting information about how long you may have to live? | Yes | No | Not Applicable | Refused | Don't know |
| --- | --- | --- | --- | --- | --- |
| 34. Not wanting to affect hope | 1 | 0 | 97 | 98 | 99 |
| 35. worried that just talking about how long you may have to live could influence how long you have to live (superstition) | 1 | 0 | 97 | 98 | 99 |
| 36. your age | 1 | 0 | 97 | 98 | 99 |
| 37. Your health | 1 | 0 | 97 | 98 | 99 |
| 38. preparing your family | 1 | 0 | 97 | 98 | 99 |
| 39. deciding on what medicines to take | 1 | 0 | 97 | 98 | 99 |
| 40. deciding whether to get tested for cancer (e.g., whether or not to get a colonoscopy when you are not having any problems) | 1 | 0 | 97 | 98 | 99 |
| 41. Your faith/religion | 1 | 0 | 97 | 98 | 99 |
| 42. wanting to accomplish life goals | 1 | 0 | 97 | 98 | 99 |
| 43. Your relationship with your primary care doctor | 1 | 0 | 97 | 98 | 99 |
| 44. Needing to be there to care for others | 1 | 0 | 97 | 98 | 99 |
| 45. Other: | 1 | 0 | 97 | 98 | 99 |

46. Please let me know if you have other thoughts about how and when your primary care doctor should talk to you about stopping cancer screening?

47. Please let me know if you have other thoughts about whether or not your primary care doctor should talk to you about how long you may have to live?

48. Please let me know if there are any other experiences you would like to share?

Thank you so much for your help.

**Clinician Questionnaire**

**We are interested in learning whether having access to information on patient life expectancy is helpful for clinical decision-making, especially when deciding when to stop recommending cancer screening. Thank you for participating in this study.**

**Perceptions of Information on patient prognosis:**

Over the past few months, we provided you with information on the life expectancy/10-year prognosis of at least one of your patients. We are interested in your reactions to this information.

1. Overall, how helpful was the information on your patient’s life expectancy/prognosis to you?

0 Not helpful

1 A little helpful

2 Somewhat helpful

3 Very helpful

2. Did you use the information to talk to any of your patients about how long they may have to live?

0 No

1 Yes

3. Was the information provided on your patient’s life expectancy/prognosis accurate from your perspective?

0 Not accurate

1 Somewhat accurate

2 accurate

4*. If not/somewhat accurate*, from your perspective did the prognostic information overestimate your patient’s life expectancy or underestimate your patient’s life expectancy/prognosis?

Overestimated by a lot

Overestimated by a little

Underestimated by a little

Underestimated by a lot

Don’t know

5. Overall, how useful was the information on your patient’s life expectancy/prognosis to you?

0 Not useful

1 A little useful

2 Somewhat useful

3 Very useful

6. Did you use the life expectancy/prognostic information to talk to any of your patients about whether or not to stop cancer screening?

0 No

1 Yes

We also sent you example language for talking to your patients about stopping cancer screening and their life expectancy. The next few questions are about these scripts.

Discussing stopping mammography screening:

|  | Strongly Disagree | Disagree | Neutral | Agree | Strongly Agree |
| --- | --- | --- | --- | --- | --- |
| 7. I would use these scripts frequently | 1 | 2 | 3 | 4 | 5 |
| 8. I found these scripts helpful | 1 | 2 | 3 | 4 | 5 |
| 9. I found these scripts useful | 1 | 2 | 3 | 4 | 5 |
| 10. I would recommend the scripts on stopping mammography screening to my colleagues | 1 | 2 | 3 | 4 | 5 |

Discussing stopping colon cancer screening:

|  | Strongly Disagree | Disagree | Neutral | Agree | Strongly Agree |
| --- | --- | --- | --- | --- | --- |
| 11. I would use these scripts frequently | 1 | 2 | 3 | 4 | 5 |
| 12. I found these scripts helpful | 1 | 2 | 3 | 4 | 5 |
| 13. I found these scripts useful | 1 | 2 | 3 | 4 | 5 |
| 14. I would recommend the scripts on stopping colon cancer screening to my colleagues | 1 | 2 | 3 | 4 | 5 |

15. What did you like about the scripts on stopping cancer screening?

16. What did you dislike about the scripts on stopping cancer screening?:

17. What suggestions do you have to improve these scripts

Discussing 10-year prognosis/life expectancy:

|  | Strongly Disagree | Disagree | Neutral | Agree | Strongly Agree |
| --- | --- | --- | --- | --- | --- |
| 18. I would use these scripts frequently | 1 | 2 | 3 | 4 | 5 |
| 19. I found these scripts helpful | 1 | 2 | 3 | 4 | 5 |
| 20. I found these scripts useful | 1 | 2 | 3 | 4 | 5 |
| 21. I would recommend these scripts to my colleagues | 1 | 2 | 3 | 4 | 5 |

22. What did you like about the scripts on discussing 10-year prognosis/life expectancy?

23. What did you dislike about the scripts on discussing 10-year prognosis/life expectancy?:

24. What suggestions do you have to improve these scripts

25. Before this study, have you ever recommended that an older patient stop being screened for cancer?

Yes

No

26. Before this study, have you ever avoided talking to a patient about stopping cancer screening because you did not want to talk about their life expectancy?

Yes

No

27. Before this study, have you ever talked to a patient about their prognosis when talking to a patient about whether or not to get screened for cancer?

Yes

No

| Please check whether you strongly agree, agree, neither, or disagree, or strongly disagree with the following statements: | Strongly Agree | Agree | Neither | Disagree | Strongly Disagree | Don’t know |
| --- | --- | --- | --- | --- | --- | --- |
| 28. Providing my older patients with information about how long they may have to live would result in my patients making more informed decisions about their medical care. | 1 | 2 | 3 | 4 | 5 | 99 |
| 29. Talking to my patients about how long they may have to live takes too much time. | 1 | 2 | 3 | 4 | 5 | 99 |
| 30. Doctors should talk to all patients aged 85 and older about how long they may have to live. | 1 | 2 | 3 | 4 | 5 | 99 |
| 31. Doctors should talk to all patients aged 75 and older about how long they may have to live. | 1 | 2 | 3 | 4 | 5 | 99 |
| 32. I am uncomfortable talking to my older patients about how long they may have to live. | 1 | 2 | 3 | 4 | 5 | 99 |
| 33. I prefer not to talk to my older patients about how long they may have to live because I do not want them to lose hope. | 1 | 2 | 3 | 4 | 5 | 99 |
| 34. I prefer not to talk to my older patients about how long they may have to live because I do not want them to think I have given up on them. | 1 | 2 | 3 | 4 | 5 | 99 |
| 35. I prefer not to talk to my older patients about how long they may have to live because I do not want to upset them. | 1 | 2 | 3 | 4 | 5 | 99 |
| 36. I prefer not to talk to my older patients about how long they may have to live because it is impossible to know how long someone may live. | 1 | 2 | 3 | 4 | 5 | 99 |
| 37. I prefer to talk to my older patients about how long they may have to live because it may help them with medical decisions. | 1 | 2 | 3 | 4 | 5 | 99 |
| 38. I prefer to talk to my older patients about how long they may have to live because it may help them plan for their future. | 1 | 2 | 3 | 4 | 5 | 99 |

Comments:

39.How do you prefer to estimate patients life expectancy? (check the box that most accurately describes how you estimate your patient’s life expectancy)

0. I do not estimate patient life expectancy

1. using their age

2. using their age and comorbidities

3. using their age and function

4. using their age and walking speed

5. using their age, comorbidities, and function

6. using their age, comorbidities, function, and walking speed

7. using prognostic calculators (e.g., using the ones on ePrognosis.org)

8. Using life tables and adjusting based on my patient’s health

9. Other: (Please describe)

40. Would you like patient prognostic information available in the electronic medical record?

0 No

1 Yes

41. Why or why not?

42. Are you familiar with ePrognois.org?

0 No

1 Yes

43. Have you used the calculators on ePrognosis.org to estimate your patient’s chance of living 10 years?

0 No

1 Yes

44. Have you found these tools helpful

0 No 1 a little 2 somewhat 3 completely

Why or why not?:

45. Have you talked to older adults with short life expectancy about stopping other medications or medical interventions?

0 No

1 Yes

Please describe your experience:

Comments:

46. Any other thoughts about talking to older adults with 5-10 year life expectancy about stopping cancer screening? Please describe:

47. Any other thoughts about talking to older adults with 5-10 year life expectancy about how long they may live?

**Background:**

48. What is your specialty?

1 Internal Medicine

2 Internal Medicine/geriatrics

3 Family practice

4 Family practice/geriatrics

5 Nurse practitioner internal medicine

6 Nurse practitioner geriatrics

7 Nurse practitioner family practice

8 Physician assistant internal medicine

9 Physician assistant family practice

10 Other:______________

49. Your age range:

1 20-29 years

2 30-39 years

3 40-49 years

4 50-59 years

5 60 to 69 years

6 70 and older

50. Your gender:

0 Female

1 Male

51. Your ethnicity

0 Hispanic 1 Not-Hispanic

52. Your race

0 White

1 African American

2 Asian

3 Other

53. How long have you been working within your profession (out of school)?

0 Less than 2 years

1 2 to 5 years

2 6 to 10 years

3 11 to 15 years

4 16 to 20 years

5 20 to 25 years

6 26 to 30 years

7 More than 30 years.

54. On average, how many patients do you see in a typical week?

5-30

31-60

61-100

101-150

151-200

>200

55. What is your average length of a patient visit right now?

10 minutes

15 minutes

20 minutes

25 minutes

30 minutes

35 minutes

40 minutes

45 minutes

60 minutes or longer

56. Approximately, what proportion of your patients are adults aged 75 and older?

0 1%

1 2-4%

2 5-9%

3 10-20%

4 21-30%

5 31-40%

6 41-50%

7 >50%

57. How much time do you typically have allotted for a visit with an adult aged 75 and older?

0 15 minutes

1 20 minutes

2 25 minutes

3 30 minutes

4 40 minutes

5 45 minutes

6 60 minutes

7 Other:______________________

Thank you!

**Chart Abstraction**

1. Date of Review _____/____/___ 2. Study ID#: ___________________________

3. PCP: ______________

4. Primary care clinic:

1 BIDMC HealthCare Associates

2 BIDMC Senior Health

3 APG (write in which one in comments):______________

5. Insurance type:

0 Private health insurance plan

1 Medicare

2 Medicaid

3 Free Care

4 Medicare + Medicaid

5 Medicare + Private (or Medex)

6 Medicare + HMO (ie 1^st^ Seniority, Secure Horizons, BC 65)

7 Medicare +Federal

8 Medicare + Free Care

9 Medicaid + Private

10 No coverage of any type

11 Unknown

6. First date that patient saw current PCP: ____/____/____

7. Date participated in Trial:

_____/_____/_____

8. Date of last screening mammogram before trial date

_____/_____/_____

9. Date of last colonoscopy before trial date

_____/_____/_____

10. If no colonoscopy, date of last fecal occult blood test before trial date

_____/_____/_____

11. If no colonoscopy, date of last sigmoidoscopy before trial date

_____/_____/_____

12. On trial date, did the PCP document talking to patient about cancer screening?

0 No

1 yes

13. *For female patients*: If yes, did the PCP document talking to patient about mammography?

0 No

1 yes

14. If yes, what did the PCP recommend?

0 to stop getting mammograms

1 to continue getting mammograms

2 to continue getting mammograms but specified how many more times

3 unknown

15. Please copy the exact text (deidentified) referring to mammography screening:

16. *All patients*: If yes, did the PCP document talking to patient about colonoscopy or other forms of colon cancer screening?

0 No

1 yes

17. If yes, what did the PCP recommend?

0 to stop getting colon cancer screening

1 to continue going for colon cancer screening

2 to continue going for colon cancer screening but for a specified time period

3 unknown

18. Please copy the exact text (deidentified) referring to colon cancer screening:

19. Did the PCP talk to patient about prognosis?

0 No

1 yes

20. If yes, please copy the text referring to talking about prognosis (Please also include any text that describes patient feelings)?

21. *For female patients*: Did the patient make a decision about whether or not to continue mammography screening?

0 No

1 Yes, patient decided to continue mammograms

2 Yes, patient decided to continue with one more mammogram and then stop

3,Yes, patient decided to stop mammography

4, other:

22. *All patients*: Did the patient make a decision about whether or not to continue colon cancer screening (e.g. colonoscopies, stool tests or fecal occult blood tests [FOBT])?

0 No

1 Yes, patient decided to continue colon cancer screening

2 Yes, patient decided to continue with one more colon cancer screening and then stop

3,Yes, patient decided to stop getting screened for colon cancer

4, other:

23. Weight:

24. Height:

25. BMI:

26. *For female patients*: History of mammography screening use:

0 Every year

1 Every other year

2 Every 3 years

3 Every 4-5 years

3 rarely

4 Never

*All patients*: History of colon cancer screening

27. Number of colonoscopies documented:_______

28. Number of sigmoidoscopies documented:_________

29. Number of fecal occult blood testing documented:_________
